# A linear presentation of cutaneous Rosai Dorfman disease

**DOI:** 10.1016/j.jdcr.2025.05.004

**Published:** 2025-06-04

**Authors:** Emily Sheetz, Ryan C. Saal, Molly K. Smith, Rachel Chikowski Byrd

**Affiliations:** a Eastern Virginia Medical School at Macon and Joan Brock Virginia Health Sciences at Old Dominion University, Norfolk, Virginia; b Pariser Dermatology, Norfolk, Virginia

**Keywords:** CD68+, cutaneous Rosai-Dorfman disease, emperipolesis, S100+

## Case description

**Fig 1:**
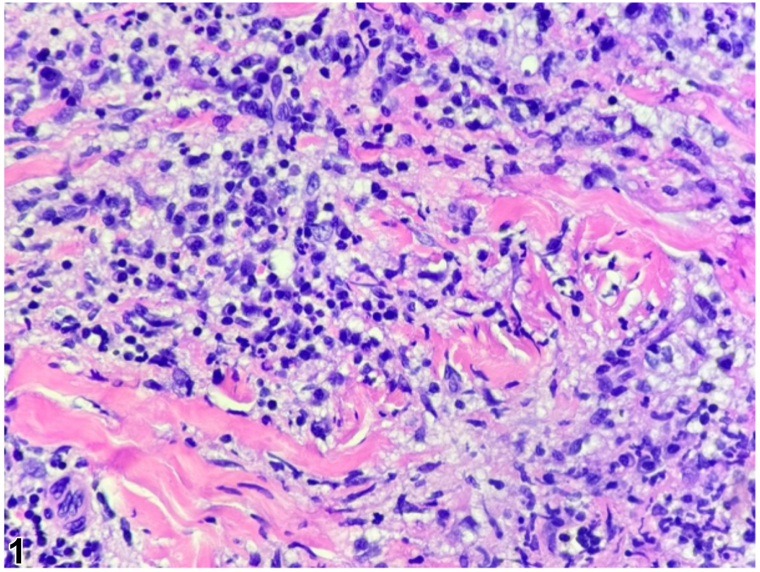

**Fig 2:**
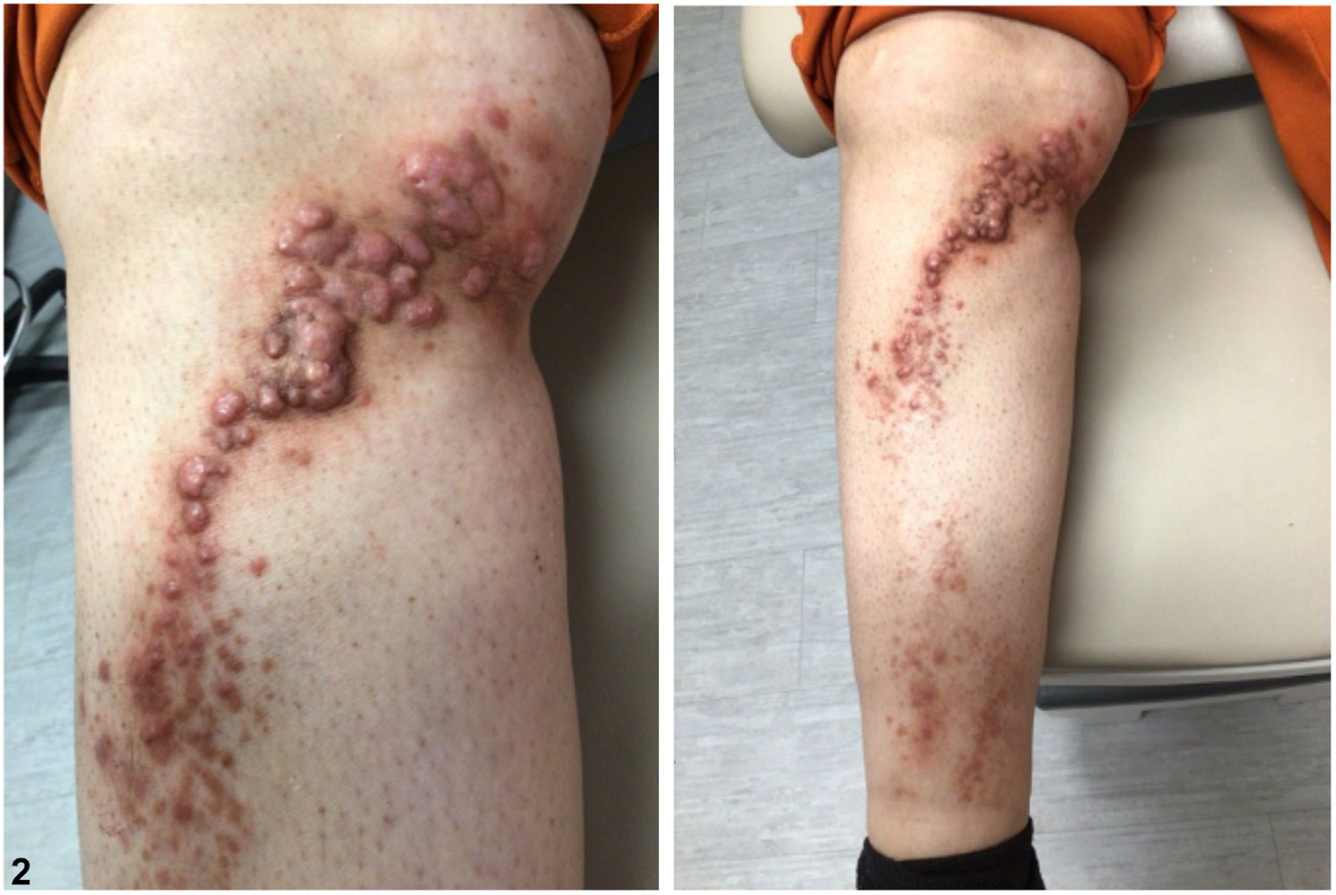

A 53-year-old female with type II diabetes and hypertension presented to the dermatology clinic with a 3-month history of an asymptomatic linear eruption along the right knee that extended distally along the shin. Initial biopsy showed an atypical lymphohistiocytic infiltrate (Fig 1), which was found to be polyclonal. At her 3-month follow up visit, the eruption demonstrated significant clinical progression with numerous indurated papules and papulonodules, some of which were tender, along the right knee and pretibial surface (Fig 2). No improvement was noted with topical triamcinolone prescribed at her initial visit.

Review of symptoms was negative for night sweats, fevers, and chills and upon further examination the patient had no lymphadenopathy. Laboratory studies revealed a microcytic anemia with a mean corpuscular volume of 75 fL, mean corpuscular hemoglobin of 22.2 pg, mean corpuscular hemoglobin concentration of 29.6 g/dL, elevated red cell distribution width of 17.3%, and thrombocytosis with a platelet count of 558 × 10^3^/μL.

An additional biopsy was performed, revealing a dense lymphohistiocytic infiltrate throughout the dermis. A CD163 stain was positive for numerous histiocytes. Larger histiocytes were present, occasionally containing multiple nuclei, confirming the presence of emperipolesis. The larger cells stained positively for S100 and CD68 and negatively for CD1a (Fig 3). Accompanying lymphocytes showed positivity for CD3 and CD20 immunostaining with occasional, scattered CD30+ cells. Lymphocytes were polytypic on kappa and lambda preparations. Acid-fast bacilli, Grocott’s methenamine silver, and gram stains were negative for infectious organisms. The histopathologic findings, together with the absence of bilateral cervical lymphadenopathy and systemic symptoms, support a diagnosis of cutaneous Rosai-Dorfman disease (CRDD). Initial workup, including physical examination, symptom review, and laboratory tests is important to assess when differentiating cutaneous-limited from systemic disease.

**Figure:**
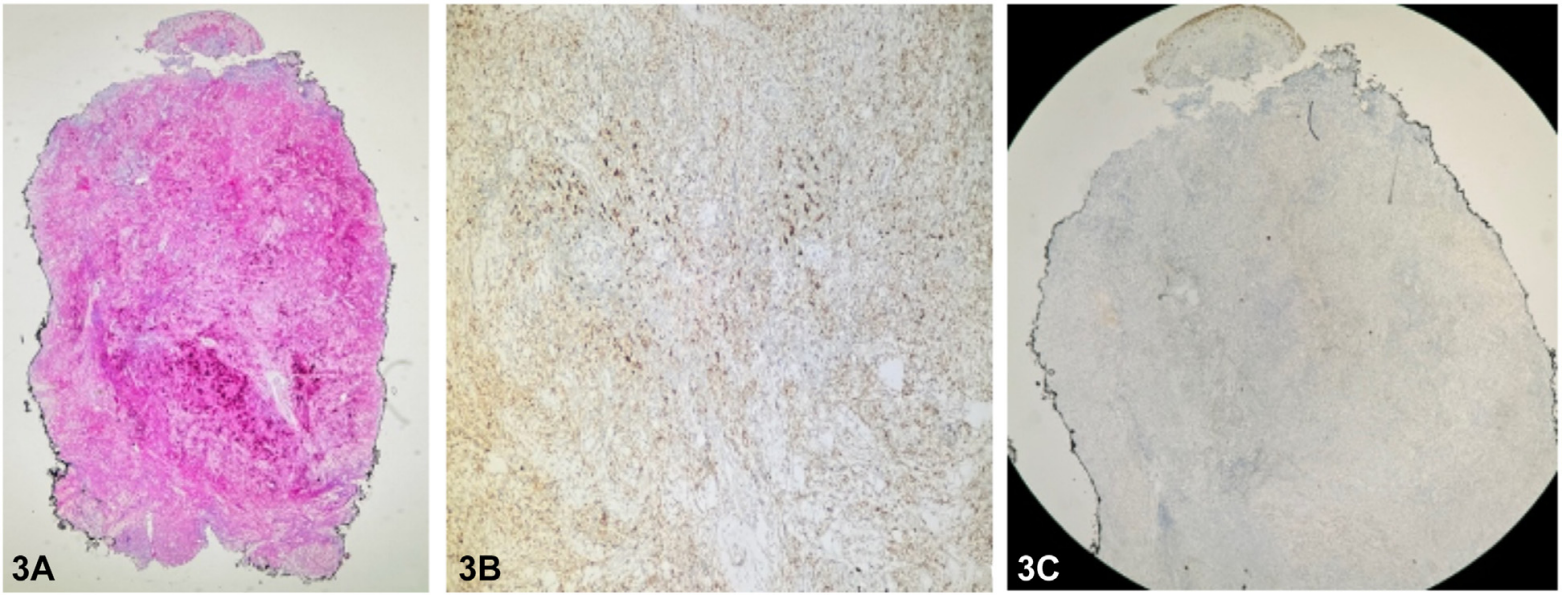

**Question 1: Which of the following clinical findings would be most suggestive of the classic form of systemic Rosai-Dorfman disease rather than the cutaneous-limited variant?**

A. Bilateral cervical lymphadenopathy
B. Isolated papulonodular eruption confined to the skin
C. Absence of fever, weight loss, or night sweats
D. Unilateral cervical lymphadenopathy
E. Histopathology of emperipolesis and S100+ histiocytes

## Discussion

Rosai-Dorfman disease is a histiocytic disorder that commonly presents with bilateral cervical lymphadenopathy and systemic symptoms such as fever and night sweats.^1^^,^^2^ Extra-nodal disease, which presents without lymph node involvement or constitutional symptoms, is rare. A category of extra-nodal disease, known as CRDD, is confined to the skin and can manifest as red-brown to yellow papules, nodules, or plaques that can be localized or diffusely scattered.^3^ In our report, we describe an unusual linear configuration of CRDD, a pattern rarely documented in the literature and one that could easily be mistaken for other linear dermatoses.

Confirmation of CRDD requires histopathologic examination to identify the presence of histiocytes that stain positively for S100 and CD68, and negatively for CD1a.^4^ These findings are shared by both systemic and cutaneous variants of Rosai-Dorfman disease and therefore are not sufficient to distinguish between them.

CRDD typically follows a benign clinical course.^5^ However, lesions may cause discomfort, and treatment should be customized based on the patient’s needs. Our patient is currently undergoing treatment with high potency topical steroids and intralesional steroid injections. Since initiating treatment, there has been noticeable improvement in the lesions, with a reduction in size and discomfort (Fig 4). This case is an example of a unique clinical presentation of CRDD, underscoring the importance in considering this condition when assessing patients presenting with a linear dermatosis.

**Figure:**
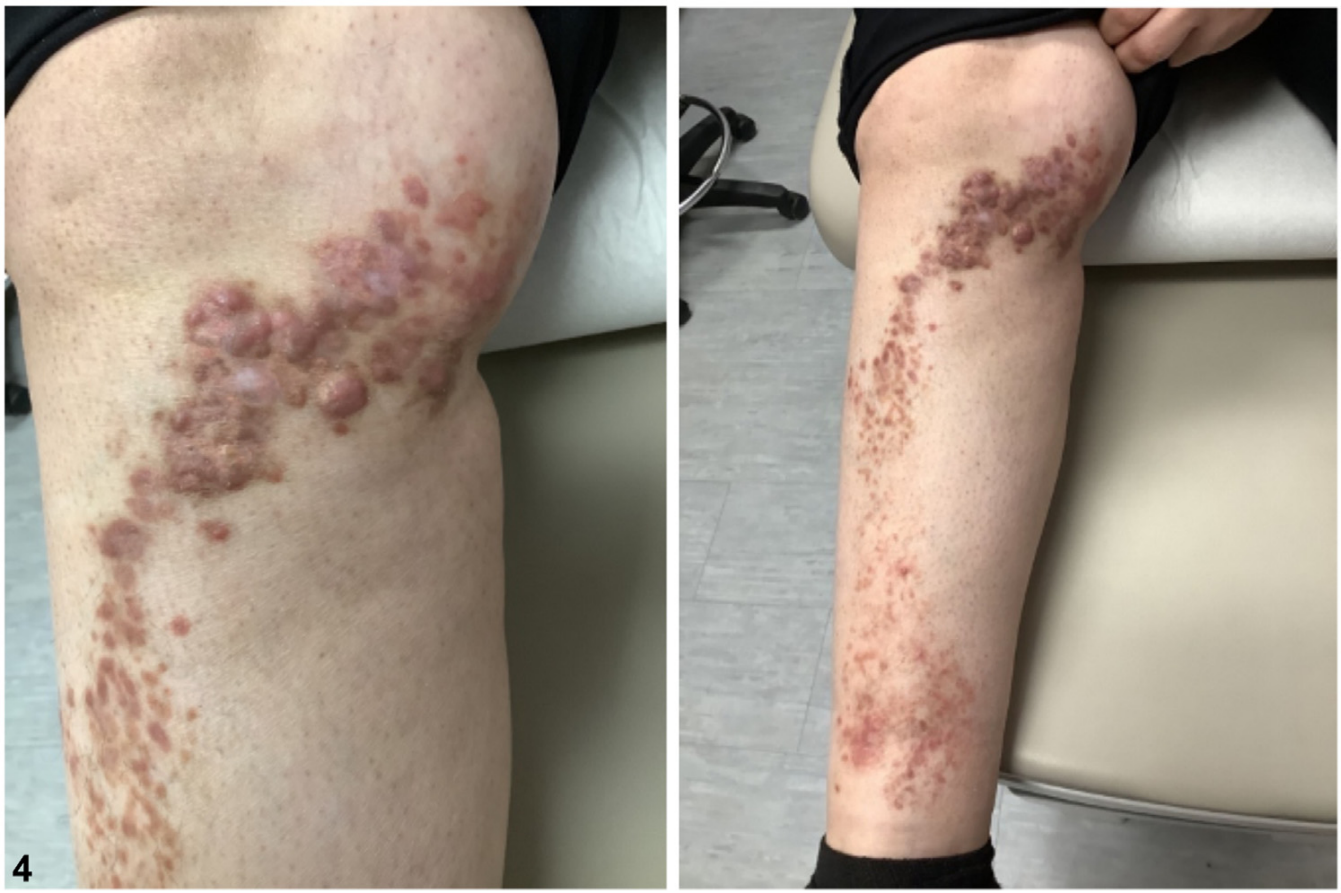

## Conflicts of interest

None disclosed.
